# The Shape of the Jaw—Zebrafish Col11a1a Regulates Meckel’s Cartilage Morphogenesis and Mineralization

**DOI:** 10.3390/jdb10040040

**Published:** 2022-09-22

**Authors:** Jonathon C. Reeck, Julia Thom Oxford

**Affiliations:** Department of Biological Sciences, Biomolecular Research Center, Center of Biomedical Research Excellence in Matrix Biology, Boise State University, Boise, ID 83725, USA

**Keywords:** Meckel’s cartilage, jaw development, mineralization, craniofacial, zebrafish, Stickler syndrome type II, Marshall syndrome, fibrochondrogenesis

## Abstract

The expression of the *col11a1a* gene is essential for normal skeletal development, affecting both cartilage and bone. Loss of function mutations have been shown to cause abnormalities in the growth plate of long bones, as well as in craniofacial development. However, the specific effects on Meckel’s cartilage have not been well studied. To further understand the effect of *col11a1a* gene function, we analyzed the developing jaw in zebrafish using gene knockdown by the injection of an antisense morpholino oligonucleotide using transgenic Tg(sp7:EGFP) and Tg(Fli1a:EGFP) EGFP reporter fish, as well as wildtype AB zebrafish. Our results demonstrate that zebrafish *col11a1a* knockdown impairs the cellular organization of Meckel’s cartilage in the developing jaw and alters the bone formation that occurs adjacent to the Meckel’s cartilage. These results suggest roles for Col11a1a protein in cartilage intermediates of bone development, the subsequent mineralization of the bony collar of long bones, and that which occurs adjacent to Meckel’s cartilage in the developing jaw.

## 1. Introduction

Jaw development requires the coordinated migration of pluripotent cranial neural crest cells (CNCC) from their site of origin at the dorsal neural tube, their proliferation, differentiation, and the subsequent orchestration of cellular adhesion, condensation, polarization, extracellular matrix production, and cellular organization that all together support the extension and the shape of the jaw. While the development of Meckel’s cartilage and formation of the vertebrate jaw have been extensively studied, the role of specific extracellular matrix components in determining the final shape and size of the jaw remains incompletely described [1,2,3].

The CNCC of the seven pharyngeal arches occupy the appropriate segmented arches through predetermined migratory streams during craniofacial development [4]. The first of seven pharyngeal arches give rise to the Meckel’s cartilage, which functions as the cartilage template of the mandible of the lower jaw [5]. Ossification of the mandible occurs through a combination of intramembranous ossification at the mediolateral dentary and endochondral ossification at the distal-most central cartilage [6].

During development, much of the skeleton goes through a cartilage intermediate, a firm yet flexible connective tissue comprising chondrocytes embedded in a collagen and proteoglycan rich matrix. Collagens are a large family of extracellular molecules of at least 29 types found mostly in connective tissues [7,8]. The functions of collagens are diverse and they play essential roles in cell adhesion, migration, structure, and cell signaling during skeletal morphogenesis [9].

The human collagen type XI alpha 1 gene, *COL11A1*, is expressed by chondrocytes in developing fetal cartilage, the growth plate, and articular cartilage. The translated protein is incorporated into collagen type XI fibrils and primarily localized to thin fibrils of the pericellular matrix of chondrocytes. Collagen type XI heterotrimeric molecules are composed of two unique alpha chains, α1(XI) and α2(XI), as well as the α3(XI) chain, which is an over-glycosylated form of collagen α1(II) [10,11]. Autosomal recessive null mutations in *COL11A1* cause the lethal chondrodysplasia, fibrochondrogenesis in humans [12]. Mutations that reduce or modify *COL11A1* gene expression cause milder forms of chondrodysplasia in Stickler’s and Marshall’s syndrome [13,14]. Fibrochondrogenesis is clinically characterized by long bone and rib shortening with flared metaphysis or cupping, vertebral malformation, flattened midface, reduced jaw protrusion, and sensorineural defects affecting hearing and vision [15,16,17]. 

Ultrastructural and biochemical analysis of collagen type XI alpha one chain molecules have provided valuable information regarding structure, location, and binding interactions [18,19,20,21]. In addition, clinical samples and animal models have indicated a clear link between chondrodysplasia, chain misassembly, and *COL11A1* gene defects [22]. In the homozygous chondrodysplasia (cho/cho) mouse, the deletion of a cytidine at position 570 with respect to the start site leads to a frameshift and downstream premature stop codon [23]. The resulting severe chondrodysplasia results in neonatal lethality were similar to fibrochondrogenesis in humans. The premature stop codon presumably leads to nonsense mediated mRNA decay, and a functional knockout of the Col11a1 alpha chain, as no protein could be detected [23]. The consequence of a *Col11a1* functional knockout does not result in a collagen type XI knockout, however, as alternative forms of the type XI collagen triple helical molecule can form, and in the absence of Col11a1 alpha chain, a type V/XI hybrid triple helical molecule forms [22]. In the absence of the alpha 1(XI) collagen chain, alpha 1(V) collagen chains trimerize with alpha 2(XI) and alpha 3(XI) chains, forming a stable type V/XI hybrid molecule [22].

This study seeks to elucidate the impact of zebrafish *col11a1a* on Meckel’s cartilage morphogenesis and cellular organization during jaw development. In our previous studies, zebrafish *col11a1a* expression was located within the developing jaw using in situ hybridization [24,25]. Using a recently developed antibody, we also identified the zebrafish Col11a1a protein within the developing jaw [26]. Based on previous findings, we investigated the effect of *col11a1a* gene knockdown on the cellular organization of chondrocytes within Meckel’s cartilage and the timing and location of mineralization of the developing jaw of zebrafish, both events that play critical roles in determining the shape of the jaw. Here, we report new information about the role of zebrafish Col11a1a in Meckel’s cartilage formation that includes the dysregulation of the columnar organization of chondrocytes within Meckel’s cartilage when *col11a1a* expression is knocked down. Further, we observed the dysregulation of mineralization of the developing jaw upon *col11a1a* knockdown. These studies provide new fundamental knowledge about the role of extracellular matrix components in jaw development.

## 2. Materials and Methods

### 2.1. Fish Care and Transgenic Lines

Vertebrate animal use was approved by the Institutional Animal Care and Use Committee (IACUC Protocols AC15-011 and AC21-011). Zebrafish embryos were obtained from ZIRC (Eugene, OR). The following lines were used in these studies; AB, Tg(sp7:EGFP) [27], and Tg(Fli1a:EGFP) [28].

### 2.2. Morpholino Injections

Gene-specific and standard control antisense morpholino oligonucleotides (AMO) were purchased from Genetools, LLC (Philomath, OR, USA). The *col11a1a* morpholino targeted the translational start site with the following sequence: 5′-GGGACCACCTTGGCCTCTCCATGGT-3′. A scrambled sequence morpholino was used as the control. Morpholinos were diluted in water and 0.05% phenol red. The morpholinos were injected at a volume of 2 nL and at a concentration of 3 ng/nL into 300 embryos for each group. 

### 2.3. Cartilage and Bone Staining

Five days post fertilization (dpf) zebrafish embryos were fixed overnight with 4% paraformaldehyde in a phosphate buffered saline (PBS). The samples were washed with PBS with 0.1% Tween-20 (PBT), then dehydrated through a 30–50–90% series of ethanol followed by an overnight incubation in Alcian blue solution of 0.1 mg/mL in 75:25% ethanol-acetic acid overnight with rocking. Samples were rehydrated through 70–50–30% ethanol series and bleached in 1% hydrogen peroxide for 3 h [29]. For vital imaging of calcification, live zebrafish were incubated in 30 mL of the zebrafish housing system water with 200 μL of 0.5% Alizarin Red (final concentration 0.003%) for 3 h. The fish were subsequently rinsed in zebrafish housing system water prior to imaging [30].

### 2.4. Imaging

Transgenic zebrafish were anesthetized in 0.016% tricaine methanesulfonate (MS-222) in system water prior to mounting in 0.6% (*w*/*v*) low melting point agarose containing MS-222. Confocal imaging was performed using a Zeiss LSM 510 Meta inverted laser scanning microscope. Alizarin Red vital stained images were collected by excitation between 530–560 nm and by monitoring emission at 580 nm. GFP transgene-expressing zebrafish images were generated by excitation at 488 and monitoring emission at 509 nm. Wide field fluorescence microscopy was performed using the EVOS Fl imaging system (ThermoFisher Scientific, Waltham, MA, USA). Alcian blue stained zebrafish were mounted in glycerol and imaged using an Olympus BX53 microscope.

## 3. Results: Characterization of Jaw Development in Zebrafish *Col11a1a* Knockdown

### 3.1. Knockdown of Col11a1a Leads to Skeletal Deformities in Zebrafish

**Figure 1:**
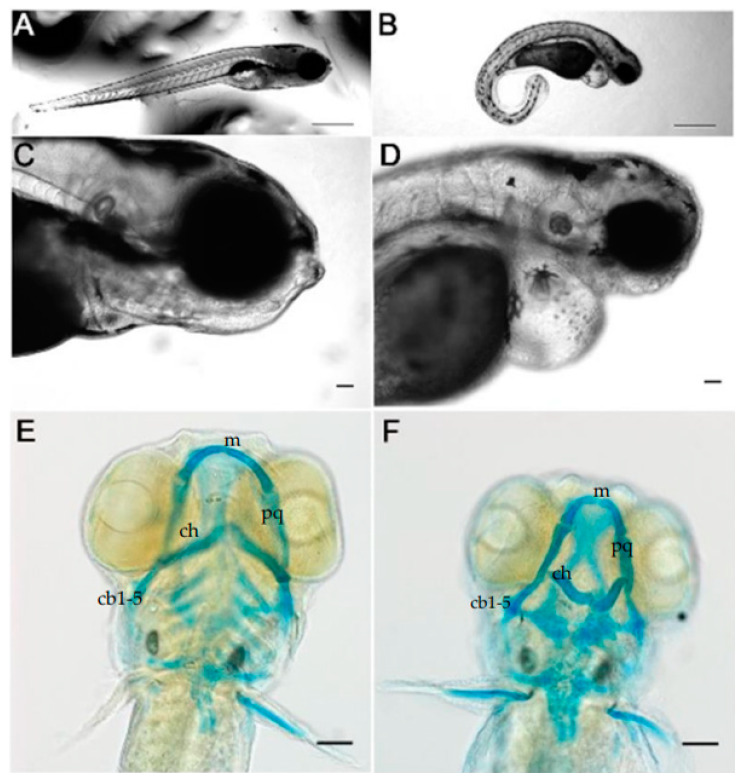
*Col11a1a* knockdown caused a curved body plan and reduced jaw size in zebrafish. Injection of a *col11a1a* targeting AMO induced a curvature of the body axis, while embryos injected with control AMO developed a straight body axis, (**A**,**B**); ((**A**,**B**) reprinted under the terms and conditions of the Creative Commons Attribution (CC BY) license (https://www.mdpi.com/2221-3759/8/3/16; Accessed on 15 July 2022) [25]. Normal face and jaw protrusion were present in the control zebrafish (**C**). In contrast, the morphant zebrafish demonstrated a reduction in face and jaw protrusion. Heart edema was also observed, (**D**). Alcian blue staining of the pharyngeal arch cartilages showed normal patterning and distally projecting cartilages (**E**), while Alcian blue staining in the morphant showed perturbed pharyngeal cartilage patterning characterized by a decreased protrusion of the 1st arch and a posteriorly facing 2nd arch, (**F**). Scale bar in (**A**,**B**) 0.5 mm; Scale bars in (**C**–**F**) 50 μm. (m; Meckel’s cartilage; pq; palatoquadrate; ch; ceratohyal; cb; ceratobranchial).

We compared morphant and wild type zebrafish at 5 dpf. This timepoint was chosen because at this time, most of the cartilage elements have developed and can be stained with Alcian blue to detect the cartilaginous ventral pharyngeal skeleton. *Col11a1a* antisense morpholino oligonucleotide (AMO) induced a curved body shape overall, making morphants distinguishable from stage-matched controls, consistent with our previous findings (Figure 1A,B) [25]. The midface had a flattened appearance and the jaw size was reduced (Figure 1C,D). In addition to the skeletal defects, heart edema was observed (Figure 1C,D), and a smaller eye size was consistently observed [31]. Upon staining of the glycosaminoglycans of the cartilage with Alcian blue, we observed that the knockdown of *col11a1a* resulted in Meckel’s cartilage and a 2nd pharyngeal arch reduction in size and altered morphology (Figure 1E,F). 

Meckel’s cartilage of the control zebrafish extended distally and lower jaw articulation was observed. In contrast, Alcian blue staining of the morphants revealed that the Meckel’s cartilage was smaller compared to stage-matched zebrafish injected with control AMO, and Meckel’s cartilage did not extend distally to the extent seen in the control. Upon further examination of the Meckel’s cartilage, we found that in addition to the reduction in distal extension, we also observed that the overall shape of Meckel’s cartilage was altered in comparison to reports from other laboratories [32,33]. Inhibition of *col11a1a* expression by injection of AMO disrupted morphogenesis of the 2nd arch, which showed a consistent bent shape, poor midline matching, and incorrectly angled ceratohyal at the midline of the zebrafish (Figure 1E,F). The remaining posterior arch structures, ceratobranchial 1–5, were more weakly stained, compared to the controls, and further lacked the proper midline angles that was observed in the control fish.

### 3.2. Cranial Neural Crest Derived Cells Form Segmented Pharyngeal Arches with Abnormal Shape

**Figure 2:**
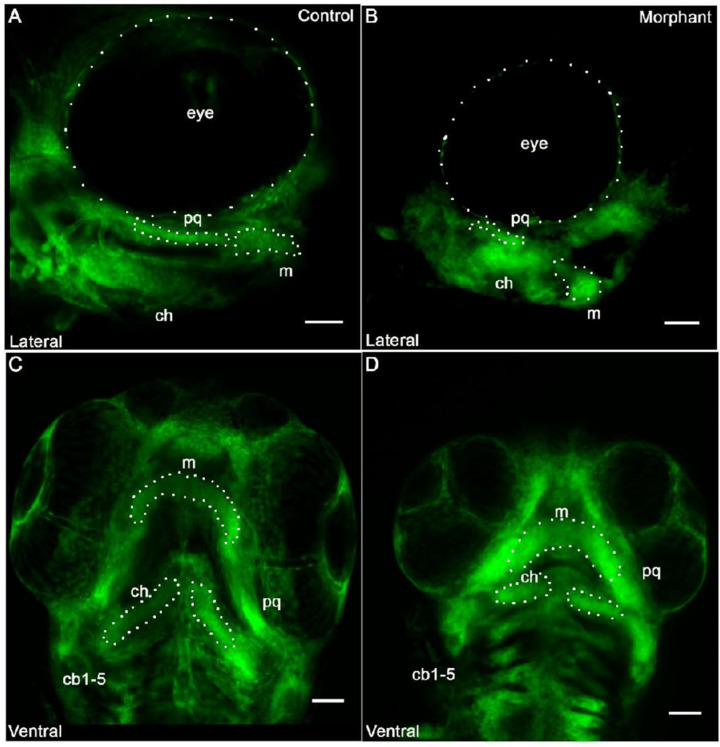
Cranial neural crest derived cells form segmented pharyngeal arches with abnormal shape under knockdown conditions of *col11a1a*. Fli1a:EGFP positive cells indicated that the neural crest derived cells were present within the pharyngeal arches. After occupying the pharyngeal arch, however, cells failed to form proper morphology. While the cells aligned to form an elongated structure in the palatoquadrate (pq) that joins the Meckel’s cartilage in the control morphant (**A**), the pq of the *col11a1a* morphant did not form the extended structure and the Meckel’s cartilage did not extend distally (**B**). The ventral view indicated segmentation of each of the developing pharyngeal arches in both the control (**C**) and the morphant (**D**). However, altered morphology was observed in the morphant (**D**). Scale bar 50 μm. (m; Meckel’s cartilage; pq; palatoquadrate; ch; ceratohyal; cb; ceratobranchial).

The cells of the pharyngeal arches were visualized by laser scanning confocal microscopy using the transgenic Fli1a:EGFP reporter (Figure 2). Fli1a is expressed in cranial neural crest derived mesenchymal cells of the developing cartilage, as well as the aortic arches and endothelial cells of the vasculature [28]. Enhanced green fluorescence protein (EGFP) expression in the neural crest derived cells of the developing pharyngeal skeletal can be detected during development [28]. We investigated the EGFP expression at 72-hpf to determine if the cells of the pharyngeal arches in morphant zebrafish were able to form the shape that is characteristic of Meckel’s cartilage, which establishes the cartilage template of the future jaw. We identified the neural crest derived structures in the morphants and compared it to the control embryos (Figure 2). We found that cells were present in the pharyngeal arches but they did not extend as observed during normal cellular organization of the developing cartilage. Neural crest derived structures formed abnormally shaped pharyngeal arches in the morphants, although the CNCCs were able to occupy segmented pharyngeal arches. However, subsequent patterning that would be consistent with normal jaw extension was not observed. Notably, the space between Meckel’s cartilage and the ceratohyal cartilage (1st and 2nd arch derivatives) was narrowed in the morphant compared to controls. Additionally, neither arch derivative extended distally to the extent seen in the controls (Figure 2B). Pharyngeal arch derivatives were present and segmented from each other, and prechondrogenic cell populations were present. However, the overall pattern and shape of these anatomical structures was not consistent with control cartilaginous structures at this stage of development.

The lateral view of the control zebrafish was used to visualize the appropriate extension of the Meckel’s cartilage at this stage of development. Meckel’s cartilage supports the lower jaw formation by forming a template for subsequent development. The morphant jaw failed to extend distally to the same extent observed in the control jaw, (Figure 2B). This appeared to contribute to both a (1) shorter and (2) wider Meckel’s cartilage. Additionally, the palatoquadrate demonstrated a wavy or bent morphology. A ventral view of the ceratobranchial arches demonstrated that the ceratobranchial arches were smaller in the morphant, when compared to the control zebrafish. The orientation of the ceratobranchial arches did not extend distally in the morphant, but instead were approximately perpendicular to the midline (Figure 2D).

### 3.3. Cells in the Meckel’s Cartilage Failed to Converge and Extend in Col11a1a Morphants

**Figure 3:**
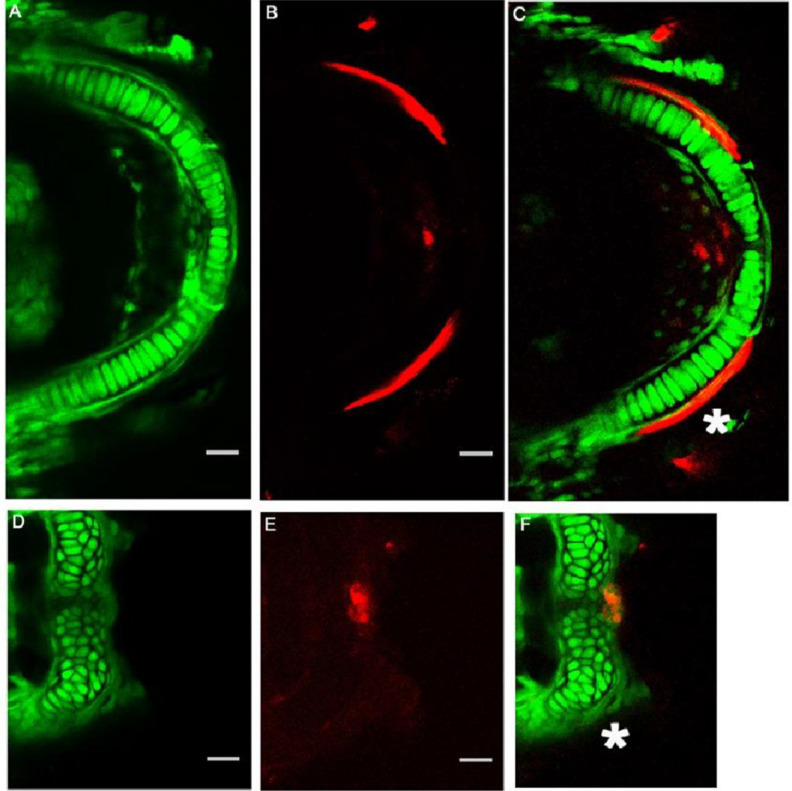
*Col11a1a* expression was required for Meckel’s chondrocyte organization, extension, and mineralization. The Fli1a:EGFP expressing cells in the Meckel’s cartilage formed an organized stack of chondrocytes that allowed the arch-like structure of Meckel’s cartilage to form (**A**) Alizarin Red staining indicated that the perichondraal cells produced a mineralized structure immediately adjacent to the Meckel’s cartilage ((**C**), white asterisk) and initiated mineralization at the central distal tip (**B**,**C**). In contrast, the cells of the morphant were not flattened, nor were they organized into a stack of cells as observed in the control ((**D**) compared to (**A**)). Lateral mineralization of the bilateral rods in the morphant was not detected ((**F**), white asterisk). Mineralization was detected by Alizarin Red staining at the central distal tip (**F**). Scale bars 20 μm.

We analyzed the cell organization of the Meckel’s cartilage in 5 dpf zebrafish to determine if the observed changes in Meckel’s cartilage shape and size correlated to the cellular organization and columnar characteristics. We observed that, while cells of the Meckel’s cartilage form a single file column of flattened chondrocytes in the control zebrafish (Figure 3A–C), in contrast, cells in the morphant failed to organize into a single stack of cells and the two bilateral rod-like arches of Meckel’s cartilage did not extend distally (Figure 3D–F).

### 3.4. Mineralization of Meckel’s Cartilage in Col11a1a Morphants Compared to Controls

We analyzed the mineralization adjacent to the Meckel’s cartilage in the *col11a1a* morphant. In the control zebrafish, we observed both cartilage forming cells, which form the single column, and perichondral intramembranous bone forming cells lining the Meckel’s cartilage. The perichondral cells produced a mineralized matrix that was detectable with Alizarin Red in controls (Figure 3B,C). In contrast, cells of the morphant failed to organize into a single stack and also did not support mineralization of the dentary, as was observed in the control fish. Some mineralization was observed at the midline, where the two rods of the Meckel’s cartilage meet at the distal-most region (Figure 3E,F). This is a site presumed to mineralize through endochondral ossification [34].

### 3.5. Sp7/Osterix Expression by Bone Forming Cells in the Morphant Meckel’s Cartilage

**Figure 4:**
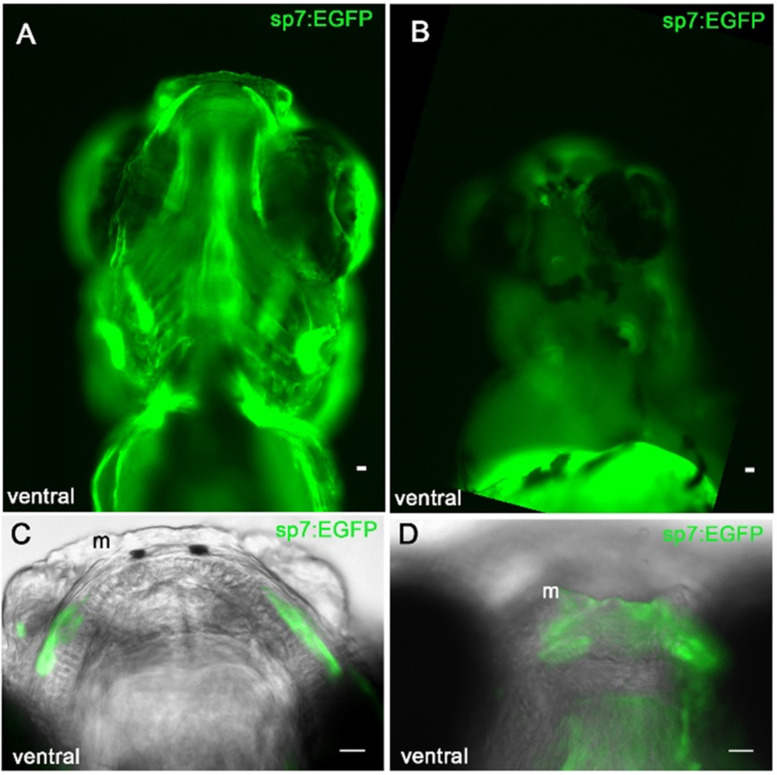
*Col11a1a* knockdown disrupts the organization of bone forming cells that line the Meckel’s cartilage. Ventral view of sp7:EGFP positive cells in 5 dpf control zebrafish (**A**,**C**) and morphant (**B**,**D**). sp7:EGFP expressing cells line the cartilage template in the control zebrafish (**C**). sp7:EGFP expressing cells form small condensations at the mediolateral cartilage but fail to occupy the location of future mineralization immediately adjacent to the Meckel’s cartilage as observed in controls (**D**); Scale bar 20 μm.

Sp7/osterix expression in bone forming cells has been reported in mice and zebrafish models [27,35]. To determine if the bone forming perichondral cells adjacent to the Meckel’s cartilage were displaced by the abnormal template shape, we tracked the location of the EGFP positive cells in the transgenic sp7:EGFP zebrafish. We investigated whether or not the osteoblasts were able to organize adjacent to Meckel’s cartilage to form the mineralized jaw. Sp7:EGFP positive cells were detected by fluorescence microscopy adjacent to the developing Meckel’s cartilage (Figure 4A). The sp7:EGFP positive cells in the control zebrafish were detected adjacent to the cartilage and the mineralization pattern followed the expression pattern of sp7:EGFP positive cells (Figure 4A). In the developing morphant jaw, however, sp7:EGFP cells were located in the mediolateral cartilage rods, but the cells did not line the cartilage as was observed in the control. Instead, the cells formed small condensations adjacent to the cartilage (Figure 4D). As shown in Figure 3, these cells did not produce detectable mineralization at this time point.

## 4. Discussion

Collagen expression is essential for normal development and altered expression has been linked to a large and diverse group of skeletal developmental diseases [36,37]. Endochondral bone formation begins when mesenchymal cell condensations form and initiate the production of the cartilage template. The cartilage increases in size and develops shape and structure through cell maturation and extracellular matrix production [38]. While *COL11A1* has been linked to endochondral ossification and to chondrodystrophies in humans, the influence of Col11a1a protein in zebrafish jaw development and intramembranous bone formation has remained unclear. Determining how a Col11a1a-deficient matrix may impact cartilage and bone morphogenesis through the spatial organization and behavior of cells is an important step in understanding skeletal development in all species, as well as developmental diseases in humans.

In this study, we analyzed the spatial arrangement of cells in zebrafish during Meckel’s cartilage development after *col11a1a* knockdown by AMO injection. Meckel’s cartilage, first described in 1820 by Johann Friedrich Meckel in human embryos, was described as a support for the jaw during development, as well as for the subsequent jawbone formation [39]. Through fluorescence microscopy and tissue staining, we determined that *col11a1a* deficiency disrupted the morphogenesis of pharyngeal arch derived cartilage and bone by preventing the normal arrangement of cells. This may be due to the changes in the extracellular matrix composition that result from reduced amounts of Col11a1a. Col11a1 has been linked to the organization of the collagenous matrix, and biomechanical properties of the resulting cartilage due to the changes in composition and organization [40].

Collagen type II is a major component of the cartilage extracellular matrix secreted by chondrocytes. Collagen type XI plays a structural role in the collagens of cartilage by nucleating and limiting the diameter of collagen type II fibrils and interacting with non-collagenous molecules [20,41]. A previous study demonstrated severe changes to the extracellular matrix and collagen network when *col11a1a* AMO was injected into zebrafish embryos [42]. *Col11a1* mutations in the chondrodysplasia (cho) mouse also showed disordered chondrocytes and collagen fibers in the growth plates of long bones [22,23]. Micro-computed tomography (μ-CT) analysis of the cho mouse model revealed alterations in the mineralization of the developing long bones, ribs, vertebrae, and skull [43]. These reports are consistent with our findings, indicating *col11a1a* is required for normal cartilage formation and the coordination of subsequent mineralization in zebrafish jaw.

The first pharyngeal arch contributes to the Meckel’s cartilage and the bones of the inner ear [2,4,44]. In zebrafish *col11a1a* morphants, the lower jaw was affected as well as the otoliths of the developing ear, supporting the idea that developmental diseases associated with *col11a1a* may be included in the group of congenital conditions referred to as First-Arch Syndromes [45]. Previous research has identified collagen gene expression, specifically *col2a1* and *col11a1a* in the developing zebrafish ear [24,46], consistent with reported craniofacial and hearing defects that occur in fibrochondrogenesis, as well as Stickler’s syndrome patients [16,47]. Fibrochondrogenesis is clinically distinguished by wide long bone metaphysis, abnormally pear-shaped vertebral bodies, flattened facial appearance, micrognathia, and abnormal curvature of the spine. In addition, ultrastructural changes in the growth plate cartilage have been observed [12,15,48].

**Figure 5:**
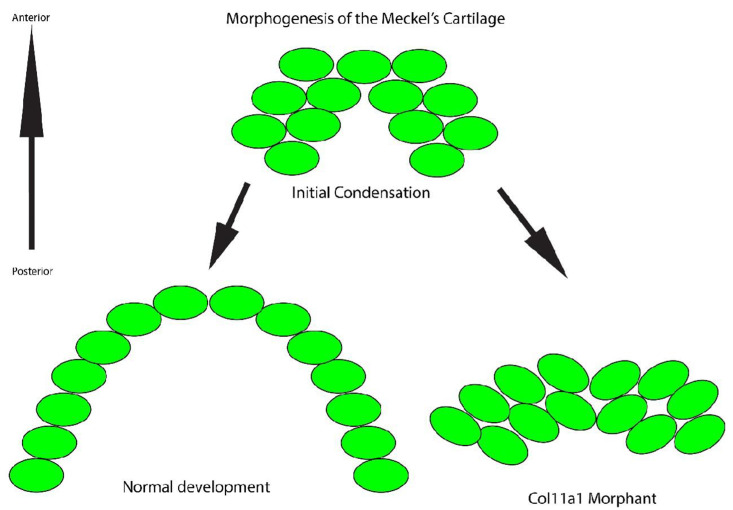
Model of Meckel’s cartilage development and the cellular organization in normal development and in the *col11a1a* morphant. During normal development, the cells (green) reorganize and form a single file stack of cells that promotes extension distally. In contrast, inhibition of *col11a1a* expression prevents reorganization into stacked cells. Therefore, the Meckel’s cartilage does not extend distally.

**Figure 6:**
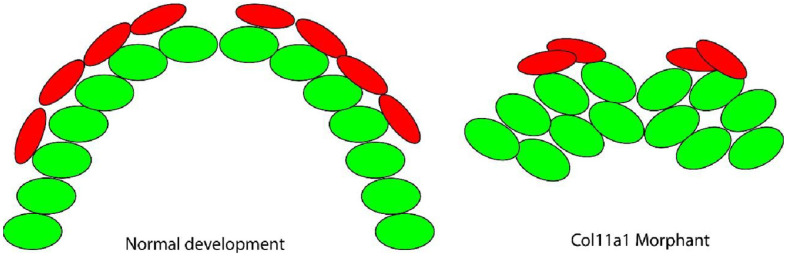
Model of Meckel’s cartilage mineralization in the *col11a1a* morphant. The organized chondrocytes (green) serve as a template for bone forming cells (red) to generate a calcified matrix. These cells extend along the template and form two bilateral mineralized rods. Therefore, perichondral cells cluster and produce abnormal mineralization at the cartilage template.

These data support the conclusion that *col11a1a* is essential to support the columnar cellular arrangement and proper timing and location of mineralization in Meckel’s cartilage. Based on the findings presented here, we suggest that (1) the loss of Col11a1a protein may prevent cell-matrix interactions that permit cell intercalation as shown in Figure 5, as well as (2) loss of Col11a1a protein may alter cartilage matrix mineralization as shown in Figure 6.

This study focused on the overall shape and morphology of cartilages, the arrangement of cells that contribute to Meckel’s cartilage and other craniofacial structures, morphogenesis, and the accumulation of proteoglycans as indicated by Alcian Blue, and the subsequent mineralization as indicated by Alizarin Red.

It is important to note that chondrodystrophies linked to *COL11A1* present as a spectrum of disorders varying in severity, such as Stickler’s syndrome [49]. Future studies will further investigate the mechanisms that underlie why the cells fail to organize properly. It is possible that non-canonical Wnt signaling and planar cell polarity programs are disrupted in *col11a1a* chondrodystrophies. The role of Col11a1a protein in cell signaling pathways that mediate planar cell polarity, as well as integrin-matrix interactions may play a role in these processes [50]. Future studies may also investigate the role of Col11a1 protein in BMP and Wnt signaling.

## 5. Conclusions

In conclusion, the loss of zebrafish *col11a1a* gene expression affects the extracellular matrix composition and organization, and has profound consequences for the spatial organization of cells during the development of the craniofacial skeleton. Mechanisms involving Col11a1a protein during Meckel’s cartilage development described here may be conserved in other examples of tissue development and degeneration, such as cancer tumor growth [51,52] and osteoarthritis [40,53]. Therefore, understanding the impact of *col11a1a* gene expression on cell behavior may contribute to elucidating the fundamental cellular mechanisms of growth, differentiation, and homeostasis of cartilage and bone.

## Data Availability

Data supporting reported results is included in this manuscript.
